# Thymic Acid

**Published:** 1868-12

**Authors:** 


Thymic Acid—Obtained from the essential oil of thyme (*thymus vulgaris*) has been proposed as a substitute for carbolic acid or creosote. It is powerfully antiseptic, and emits no disagreeable odor. In its concentrated form, it may take the place of nitrate of silver as an escharotic; as an antiseptic it should be dissolved in 100 parts of water with a little alcohol.—*Humboldt Medical Archives.*

